# The Associations between the Family Education and Mortality of Patients on Peritoneal Dialysis

**DOI:** 10.1371/journal.pone.0095894

**Published:** 2014-05-05

**Authors:** Zhi-Kai Yang, Qing-Feng Han, Tong-Ying Zhu, Ye-Ping Ren, Jiang-Hua Chen, Hui-Ping Zhao, Meng-Hua Chen, Jie Dong, Yue Wang, Chuan- Ming Hao, Rui Zhang, Xiao-Hui Zhang, Mei Wang, Na Tian, Hai-Yan Wang

**Affiliations:** 1 Renal Division, Department of Medicine, Peking University First Hospital, Institute of Nephrology, Peking University, Key Laboratory of Renal Disease, Ministry of Health, Key Laboratory of Renal Disease, Ministry of Education, Beijing, China; 2 Department of Nephrology, Peking University Third Hospital, Beijing, China; 3 Department of Nephrology, Huashan Hospital of Fudan University, Shanghai, China; 4 Department of Nephrology, Second Affiliated Hospital of Harbin Medical University, Heilongjiang, China; 5 Kidney Disease Center, The First Affiliated Hospital, College of Medicine, Zhejiang University, Hangzhou, China; 6 Department of Nephrology, Peking University People's Hospital, Beijing, China; 7 Department of Nephrology, General Hospital of Ningxia Medical University, Ningxia, China; Iran University of Medical Sciences, Iran (Islamic Republic Of)

## Abstract

**Aims:**

To investigate whether education level of family members predicts all-cause and cardiovascular death and initial-episode peritonitis in patients on peritoneal dialysis (PD).

**Methods:**

A total of 2264 patients on chronic PD were collected from seven centers affiliated with the Socioeconomic Status on the Outcome of Peritoneal Dialysis (SSOP) Study. All demographic, socioeconomic and laboratory data of patients and the education level of all family members were recorded at baseline. Multivariate Cox regression was used to calculate the hazard ratio (HR) of all-cause and cardiovascular mortality, and initial-episode peritonitis with adjustments for recognized traditional factors.

**Results:**

There were no significant differences in baseline characteristics between patients with (n = 1752) and without (n = 512) complete education information. According to the highest education level of patients' family, included 1752 patients were divided into four groups, i.e. elementary or lower (15%), middle (27%), high (24%) and more than high school (34%). The family highest education (using elementary school or lower group as reference, hazard ratio and 95% confidence interval of middle school group, high school group and more than high school group was 0.68[0.48–0.96], 0.64[0.45–0.91], 0.66[0.48–0.91], respectively) rather than their average education level or patients' or spouse's education was significantly associated with the higher mortality. Neither patients' nor family education level did correlate to the risk for cardiovascular death or initial-episode peritonitis.

**Conclusions:**

Family members' education level was found to be a novel predictor of PD outcome. Family, as the main source of health care providers, should be paid more attention in our practice.

## Introduction

Peritoneal dialysis (PD) has been utilized as one of the main renal replacement therapies since the 1980s. Although the number of PD patients has markedly increased in both developing and developed countries[1], PD outcomes, such as mortality, technique failure, and hospitalization, have not markedly improved. Potential risk factors for poor outcome have been continuously explored in recent years. Among these, socioeconomic status (SES) has been indicated as a key predictor through multi-center PD cohort studies from various countries. These studies indicate that SES evaluated by individual education[2] and income[3], housing status[4], remote location[5], [6], or social support[7]–[11] play the critical role in the outcomes of dialysis patients. Based on the inverse relationship between individual SES and mortality from our large-scale multi-center retrospective PD cohort study[3], we would further explore the association of social support and PD outcome.

Social support is the intricate network in which patients with various chronic illnesses may give and receive information and aid and have emotional needs met[12], which is mainly sourced from family members including a spouse, children and relatives, friends, and colleagues. For patients who receive long-term home care therapy, family members are the most important healthcare providers. At the start of dialysis, family members the main drivers for choosing PD or hemodialysis (HD) as a treatment[13], [14]. Family members are also involved in the accommodation of lifestyle and living environment changes, and helping patients to improve their compliance to a therapy regime. For elderly or disabled patients, family members are more likely to take more responsibility for PD-associated care, such as performing the PD exchange and exit-site care, monitoring symptoms and signs daily, and contributing to food preparation and nutrition provision. All of the above are dependent on a strong education base[15]. Hence, it is hypothesized that the education level of family members may play a key role in the quality of therapy and PD outcome. To date, social support as a general index of SES rather than family members' education status has been investigated with respect to its impact on dialysis outcome in previous studies[7]–[11].

Therefore, we aimed to investigate associations between education level of PD patients' family members and outcome events, including all-cause and cardiovascular death and first-episode peritonitis through a large-scale multi-center retrospective cohort study, which will be helpful for unpacking the black box of the family education-outcome puzzle for PD population.

## Methods

This is an affiliated study with the Socioeconomic Status on the Outcome of Peritoneal Dialysis (SSOP) study, which is a retrospective multi-center cohort study as described in detail in our previous paper[3]. The ethics committee of Peking University First Hospitl, China approved this study. Written consent was given by the patients for their information to be stored in the hospital database and used for research.

### Center Enrollment

Centers with professional PD doctors and nurses and well-developed databases maintained for least 3 years, recording baseline characteristics and follow-up data every 1 to 3 months, participated in this study voluntarily. Nine centers were qualified, and seven of these, accounting for about 70% of all incident patients attending the nine centers, agreed to participate. The included PD centers were located in five different provinces and four geographical regions (north, northeast, northwest, and east) of China. Data from each center were collected within a strict quality control framework and further inspected and optimized to ensure the integrity and accuracy of the database. All study investigators and staff members completed a training program that taught them the methods and processes of the study. A manual of detailed instructions for data collection was distributed.

### Subject Selection

All incident patients receiving chronic PD between the date of intact database creation and August 2011 were enrolled into this study. After starting PD, each patient signed informed consent agreeing to the use of their demographic and laboratory data in future studies. Those without information of education levels of family members were excluded. All subjects began the PD program within 1 month after catheter implantation and were given lactate-buffered glucose dialysate with a twin-bag connection system (Baxter Healthcare, Guangzhou, China).

### Data collection

Demographic and clinical data including age, gender, body mass index (BMI), primary renal disease, history of cardiovascular disease (CVD), and presence of diabetes mellitus (DM) were collected at baseline. CVD was recorded if one of the following conditions was present: angina, class III/IV congestive heart failure (New York Heart Association), transient ischemic attack, history of myocardial infarction or cerebro-vascular accident, or peripheral arterial disease[16]. Baseline biochemistry data including hemoglobin, serum albumin, calcium, phosphate and intact parathyroid hormone (iPTH) were examined using an automatic Hitachi chemistry analyzer and then calculated as the mean of measurements made during the first 3 months. Dialysis adequacy and residual renal function (RRF) were measured during the first 6 months. RRF was defined as the mean of residual creatinine clearance and residual urea clearance. Dialysis adequacy was determined from the total Kt/V and total creatinine clearance (Ccr). Center size was also recorded according to the number of enrolled patients from each center.

The education level of patients and each family member, including spouses of those who are married was recorded from 1 to 4 as ordinal categorical variables according to diploma obtained based on school level: elementary school or lower  = 1; middle school  = 2; high school  = 3; and more than high school  = 4. Average education of a whole family except for the patient was calculated as the arithmetic mean of the education levels of all family members. The highest education of any one family member was recorded as the maximum education level of family members.

Family income was defined as the yearly household income per person and was divided into low (<¥20,000, <$3160,), medium (¥20,000–40,000, $3160–6320) and high (>¥40,000, >$6320) according to average income for urban information in 2011 from the bureau of statistics (http://www.bjstats.gov.cn/nj/main/2011-tjnj/index.htm) since most subjects were from urban. Information of reimbursement type and family residence was also collected. The frequent visitor was defined as someone visiting doctors at least one time every 3 months. Whether medical expenses are covered by national health care system was also recorded

### Definition of Outcome Events

The Primary outcome was defined as all-cause death and cardiovascular death. Cardiovascular death was defined as death due to myocardial infarction, congestive heart failure, cerebral bleeding, cerebral infarction, arrhythmia, peripheral arterial disease, and sudden death. The secondary outcome was initial peritonitis, which was diagnosed according to International Society for Peritoneal Dialysis 2010 guidelines[17]. In all analyses, data of transferring to hemodialysis (HD), loss to follow-up, renal transplantation or till the end of the study (November 1, 2011) were censored.

### Statistical Analysis

Continuous data were presented as mean with standard deviation except that RRF was presented as the median (inter-quartile range) because of high skew. Categorical variables were presented as proportions. Relevant characteristics were compared between different education groups, respectively. Patient data were compared using the one-way ANOVA for normally distributed continuous variables, or the Kruskall–Wallis H test for skewed continuous variables, and the Chi-square test for categorical variables. Spearman correlations were explored to identify correlation among various indices of education level and family income. To determine predictive effect of education level of the patient and family (couple education, average education and the highest education) on the outcome events, stratified multivariable Cox regression models were explored, adjusted by age, gender, BMI, presence of DM, CVD history, baseline albumin, hemoglobin, RRF, and family income, and center size was as the stratified factor to adjust for center effects. The center effect was reflected not only in disparity of center size (ranging from 78 patients to 815 patients), which has been demonstrated to be an independent predictor of PD outcome[18]–[20], but also in differences in practice patterns and biochemical assays between centers. We reported the multivariable adjusted hazard ratios (HRs) with 95% confidence interval (CI). All probabilities were two- tailed, and the level of significance was set at 0.05. Statistical analyses were performed using SPSS for Windows software version 15.0 (SPSS Inc., Chicago, IL).

## Results

### Baseline characteristics

Data from 2,264 patients were collected. Five hundred and twelve patients were excluded due to missing family education data. In the final cohort, the included 1,752 patients had a mean age of 57.93±15.30 years, with male patients accounting for 49.9%. Overall, 38.8% were diabetic and cerebrovascular disease (CVD) was present in 43.4% of subjects at baseline. The total follow-up duration was 27.6 (14.3–45.4) months. Chronic glomerulonephritis (CGN) was the most common cause of end-stage renal disease (ESRD) (34.4%), followed by diabetic nephropathy (29.7%) and hypertensive nephropathy (16.4%). There were no significant differences in age, gender, body mass index (BMI), or distribution of education level of patients and their family members between the included and excluded subjects (*P*>0.05).

### Education levels of PD patients and their family members

**Figure 1:**
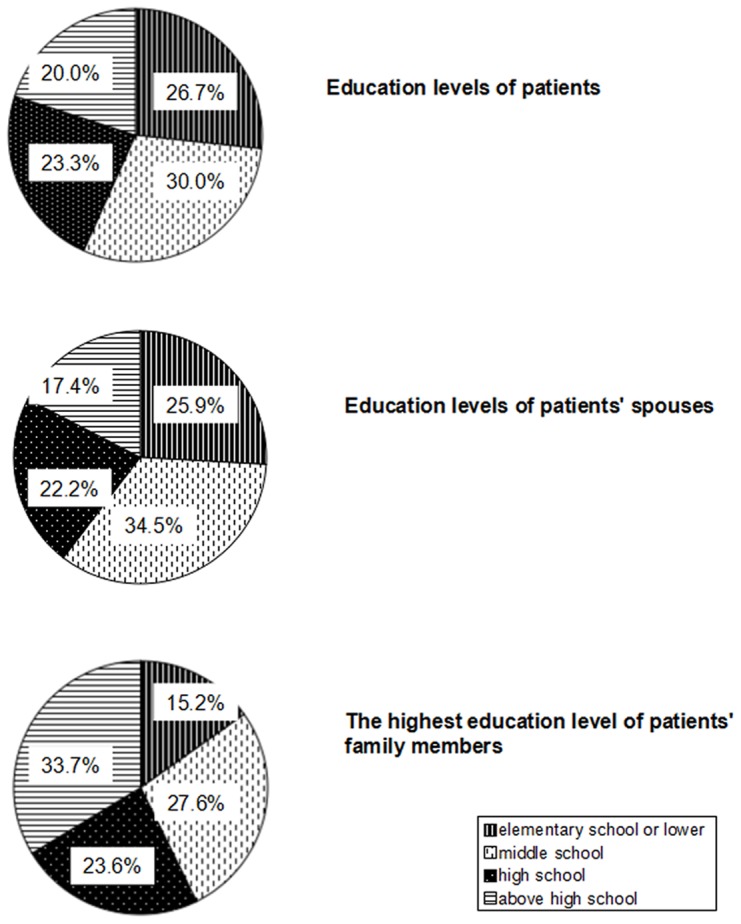
Education levels of PD patients and their family members.

The constitutions of education levels of the patients and their partners were nearly equivalent; e.g. elementary school 26.7% and 25.9%, middle school 30.0% and 34.5%, high school 23.3% and 22.2%, and more than high school 20.0% and 17.4%, respectively. As for the highest education level of the family members, 33.7% had more than high school level (**Figure 1**).

Comparing patients according to their family's highest level of education showed significant differences in age (over 65 years or not), gender, education and income level, reimbursement type (healthcare or not), or the percentage of rural residence (*P*<0.001 or 0.05; **Table 1**). Patients whose family's highest education was elementary or lower were more likely to be the eldest male patients from a rural area. They were also less likely to be covered by national healthcare, and had lower family income and lower education level. These patients also had the lowest plasma albumin at baseline. There were no significant differences in the presence of diabetes mellitus (DM), CVD history, percentage of frequent visitor, total Kt/V and Ccr, RRF, BMI, or hemoglobin (*P*>0.05).

**Table 1:** The baseline characteristics and clinical data of PD patients according to their family's highest education level.

	The highest education level of patients' family members					
	elementary school or lower		middle school	high school	above high school	*P*
**Age>65 yrs** (%)	50.38		38.30	29.30	38.47	<0.001
**Male**(%)	55.26		52.80	47.22	46.95	0.048
**BMI** (Kg/m2)	22.63±3.88		22.93±3.51	23.05±3.59	22.81±3.39	0.47
**DM** (%)	39.02		40.22	36.39	39.18	0.70
**CVD** (%)	43.68		39.28	41.83	47.20	0.06
**Family income**						<0.001
Low (%)	61.89		53.11	48.54	34.92	
Medium (%)	30.57		38.59	39.08	34.92	
High (%)	7.55		8.30	12.38	30.15	
**Education levels of patients**						<0.001
Elementary school or lower (%)	53.01		27.44	21.31	18.00	
Middle school (%)	27.82		41.37	28.33	22.75	
High school (%)	12.03		22.25	32.93	22.54	
Above high school (%)	7.14		8.94	17.43	36.67	
**Education levels of patients' spouse**						NA
Elementary school or lower (%)	100.00	14.14		12.39	12.03	
Middle school (%)	0.00	85.86		18.13	17.15	
High school (%)	0.00	0.00		69.49	17.15	
Above high school (%)	0.00	0.00		0.00	53.67	
**Average education level**	1.00±0.00	1.88±0.22		2.65±0.44	3.38±0.63	<0.001
**Rural residence** (%)	34.2	23.8		21.1	8.6	<0.001
**Health care** (%)#	59.4	68.7		73.5	84.1	<0.001
**Frequent visitors** (%)□	85.71	88.54		86.68	90.15	0.19
**Hemoglobin** (g/L)	98.76±18.16	101.82±19.39		101.45±17.51	101.52±17.16	0.07
**Serum albumin** (g/L)	34.32±5.76	34.80±5.21		35.45±5.25	35.29±5.13	0.023
**Calcium(mmol/L)**	2.16±0.25	2.17±0.23		2.19±0.25	2.20±0.24	0.08
**Phosphate(mmol/L)**	1.53±0.47	1.58±0.50		1.54±0.46	1.56±0.42	0.36
**iPTH (pg/ml)**	179.2(78∼343)	180.9(93.05∼327.15)		192(87.7∼338.5)	148(64.57∼313.15)	0.07
**Total Kt/V**	2.03±0.66	2.05±0.63		2.12±0.67	2.02±.64	0.13
**Total Ccr (L/w/1.73 m^2^)**	77.25±41.43	77.9±47.99		76.9±30.9	74.78±29.9	0.64
**RRF** (ml/min)	3.02(1.30–5.62)	3.30(1.76–5.40)		3.72(1.94–5.57)	3.51(1.93–5.50)	0.26

Abbreviations: BMI, body mass index; DM, diabetes mellitus; CVD, cardiovascular disease; iPTH, intact parathyroid hormone; Kt/V, urea clearance; Ccr, creatinine clearance; RRF, residual renal function.

# Healthcare % represents the percentage of patients whose medical expenses are covered more than 90% by the national healthcare system.

□ Frequent visitor was defined as someone visiting doctors at least once every 3 months.

Education levels of patients, their spouses, and the average and highest level of education of their family members, had positive correlations (*r* = 0.241∼0.695; *P*<0.001 for all). Likewise, each education level was correlated with yearly personal income (*r* = 0.241∼0.265; *P*<0.001 for all; **Table 2**).

**Table 2:** Coefficient correlations between the education levels of PD patients and their family members and family income^#^.

	Patients' education	Spouse's education	The highest education	Average education	Family income
**Patients' education**	NA				
**Spouse's education**	0.566	NA			
**The highest education**	0.335	0.695	NA		
**Average education**	0.428	0.825	0.874	NA	
**Family income**	0.265	0.281	0.241	0.262	NA

Abbreviation: NA, non analysis.

# All *P* values for the correlation analyses were <0.001.

### Follow-up and outcomes

Among the 497 patients who died, 190 deaths (38.2%) were due to CVD and 120 (24.1%) infection; other causes were malignancy, gastrointestinal bleeding, malnutrition, miscellaneous, and undefined. One hundred and forty-eight patients were transferred to HD, most due to PD-associated infection (67 cases, 45.3%).

The time to first-episode peritonitis was 20.88 (9.73–35.23) months. Among 392 episodes of initial peritonitis during the study period, there were 139 episodes (35.5%) due to Gram-positive organisms, while 84 (21.4%) were due to Gram-negative organisms and 7 (1.8%) fungi.

### Associations of education levels with outcome

By multivariable Cox regression analysis (**Table 3**), similar to our previous report[3], patient education level did not predict patient survival after adjusting for age, gender, BMI, presence of DM, CVD history, baseline albumin, hemoglobin, RRF, and family income, and using center size as a stratified factor to adjust for center effects. However, the highest education of family members was significantly associated with higher mortality. As compared to the family's highest education of elementary school or lower group, middle, high and more than high school education decreased the risk of death by 32%, 36%, and 34%, respectively. There also a trend that each increase of 1 in average education level decreased mortality by 11% (*P* = 0.05). As for spouse's education, middle education rather than high school or more than high school also predicted a lower mortality. For cardiovascular death and initial-episode peritonitis, neither PD patients nor their family members' education level were associated with the higher risk after adjusting for the abovementioned covariates. No interactions between education level and age, gender, DM, or CVD history were found for predicting all-cause mortality, CV death, or initial-episode peritonitis.

**Table 3:** The predictive role of education level of patients and their family members by multivariable Cox regression analysis.

	Death					Cardiovascular death				Initial- episode peritonitis			
	HR		95% CI		*P*	HR	95% CI		*P*	HR	95% CI		P
Patients' education													
Low	ref				0.47	ref			0.95	ref			0.85
Middle	0.81	0.61		1.09	0.17	0.93	0.57	1.50	0.77	0.93	0.69	1.25	0.63
High	0.87	0.63		1.21	0.40	0.88	0.52	1.49	0.64	0.91	0.66	1.27	0.59
>high	1.00	0.71		1.41	0.99	0.85	0.48	1.52	0.59	0.86	0.60	1.24	0.86
The highest education													
Low	ref				0.04	ref			0.49	ref			0.26
middle	0.68	0.48		0.96	0.03	0.74	0.42	1.33	0.31	0.85	0.58	1.26	0.43
High	0.64	0.45		0.91	0.01	0.62	0.34	1.13	0.12	1.08	0.74	1.59	0.67
>high	0.66	0.48		0.91	0.01	0.75	0.44	1.29	0.30	1.14	0.79	1.64	0.49
Spouse's education													
Low	ref				0.07	ref			0.18	ref			0.06
middle	0.69	0.49		0.97	0.03	0.69	0.41	1.17	0.17	0.90	0.62	1.29	0.56
High	0.72	0.49		1.07	0.10	0.62	0.33	1.17	0.14	1.39	0.95	2.05	0.09
>high	0.98	0.67		1.43	0.92	1.08	0.60	1.97	0.79	1.19	0.79	1.81	0.41
Average education	0.89	0.78		1.00	0.05	0.94	0.77	1.14	0.52	1.07	0.95	1.22	0.28

Notes:All models are adjusted for age, gender, BMI, presence of DM, CVD history, baseline albumin, hemoglobin, RRF and family income, and center size was used as a stratified factor to adjust for center effects.

The HRs and 95%CI for all-cause death, cardiovascular death, and initial-episode peritonitis are shown respectively using low level (elementary school or lower) as the reference. The HR and 95% of average education level as a continuous variable is also shown.

## Discussion

In this large-scale multi-center retrospective study, the highest educationof family members were identified as independent predictive factors of PD outcome; however, no such effects were detected for CV death or occurrence of the first episode of peritonitis after adjustment for well-recognized confounders. The education level of a PD patient was not found to contribute to any outcome events, similar to our previous reports[3]. To our knowledge, this is the first study to offer insight into the impact of family members' education status on PD patient outcomes.

Most patients with chronic kidney disease will develop ESRD and sooner or later require renal replacement therapy. This is unpleasant and has a great impact on both the patients and their family members. Undoubtedly, the patients' illness affects the physical and mental health of families in this population[21]–[23]. On the other hand, family members as a primary source of social support can make great contributions to patients' disease management. To date, only a few studies have shown that spouse's behavior and attitude can affect patient management of diabetes[24], [25], stroke[26], and osteoarthritis[27] rehabilitation therapy. For the dialysis population, although previous studies have indicated the significant impact of social support on satisfaction, psychological status, quality of life, hospitalization, and mortality for PD and HD patients[7]–[11], the association between a specific aspect of family members, such as education status and PD outcome has rarely been explored.

Our novel findings presented here revealed a close relationship between education level of family members and poor PD outcome. There are several reasons for these results. Firstly, for patients with ESRD, medical decisions are largely dependent on consultation with family members. Family members with high education backgrounds are more likely to get access to healthcare and offer better information sharing and advice. Such good social support from family probably leads to early referral to nephrologists, appropriate choice of dialysis modality, and timely preparation with dialysis access[28]. Secondly, once a dialysis program starts, the whole family has to strive to adapt to the changes in their daily lives, including dietary, complex medication regimens, some social isolation, frequent clinic visits, and hospitalization. Care giving activities, including appraising, advocating, and coaching, provided by family members is dependent on a strong knowledge base[15]. Family members with higher education levels can understand all the changes easily and are apt to adjust patients' lifestyles as suggested. As shown in a previous study, there is a very strong correlation between coherence and functionality of the non-chronically-ill spouse, social support, and compliance to chronic illness to his or her situation[29]. Besides the family-unit therapy nature of PD, according to our practice, deep-rooted family conception of Chinese patients may reveal the unnegligible affection from the whole family to the patient, hence patients' own education levels seemed less important. However, there was not such a linear trend that the family highest education level of middle school and above would be more benefit for patients' mortality. Though educated family would be expected to have better adherence to treatment and adaptation to daily life change, they also bear heavier mental pressure and endure more fierce decision conflict when accidents happen. Leadership, the role most likely played by person with the highest family education, might be important at such a difficult point to decision making, which would affect patients' outcomes.In addition, although close relationships among family's education level, income level, rural residence, and less healthcare were found, multivariate analysis could not validate that the predicting role of family's education level is confounded by these factors. Future studies should focus on the effect of improved social support from family members on the physical or psychosocial well-being of patients[30].

It is interesting that higher education level of the spouse did not decrease the death risk for PD patients despite the fact that the spouse has the closest relationship with patients acting as a caregiver, confidant, and primary source of emotional support. The cause for this phenomenon is not clear. However, recent studies have suggested that the quality of marriage rather than the presence of a marriage significantly influences the outcome for dialysis patients. Low patient marital satisfaction evaluated by the Dyadic Adjustment Scale is found to be associated with poorer psychological status of the partners and higher mortality[31]. Whether or how the education level of a spouse correlates to the quality of marriage and family support needs to be explored.

Based on our data, the education level of patients and their family members has no impact on initial-episode peritonitis. One possible explanation for this is that selected centers were equipped with professional PD clinicians and well-developed training programs[32]. In most centers, patients and their homecare helpers were often and repeatedly trained and examined for proper techniques with respect to bag exchange. Under these circumstances, it is likely that biological factors leading to the occurrence of peritonitis may outweigh the effects of education level of patients and their family members.

This study has several strengths. First, this is the only study to disclose the impact of education levels of both PD patients and their family members on PD outcomes with large sample sizes. The detailed information on the education status offers us a valuable chance to reveal a family education-outcome relationship. Our results alert clinicians to highlight the importance of family factors on the therapeutic effects of family-based treatment, such as in PD. In addition, the baseline education level is a fixed variable for the majority adult patients and their family. As compared to social support, a variable index during long-term follow up, baseline education status as a potential prognostic factor is more appropriate to be explored.

There are some limitations to this study. First, potential mechanisms for the association of family members' education and PD outcome are not clear since we did not evaluate a patient's satisfaction with care providers, perception of accessibility of care or to therapy for patients from less-educated families. Until such research is conducted, a determination of whether and how family members' education directly affects quality of therapy and PD outcome is difficult to make. Secondly, we should be aware of the possibility of residual confounding and recall bias because of the retrospective nature of this study. However, if confounding occurred, it would result in underestimation of the association but not change our main findings at all. In addition, we should be aware of the possibilities of ascertainment bias (totally 22.6% eligible patients were not included). Finally, as an observational study, a cause-effect relationship could not be established.

In conclusion, PD patients with well-educated family members have a lower death risk. This novel finding is helpful for us to understand the key role that family support plays in the quality of PD therapy, such a home-based therapy. Further research focusing on family-unit therapy and individualized care based on family education background should be designed to investigate their potential benefits for PD patients. The issues raised by the present study also highlight some challenges for caring practices in home-based therapy for conditions like PD.
